# Epitope Identification and Application for Diagnosis of Duck Tembusu Virus Infections in Ducks

**DOI:** 10.3390/v8110306

**Published:** 2016-11-10

**Authors:** Chenxi Li, Junyan Liu, Wulin Shaozhou, Xiaofei Bai, Qingshan Zhang, Ronghong Hua, Jyung-Hurng Liu, Ming Liu, Yun Zhang

**Affiliations:** 1 State Key Laboratory of Veterinary Biotechnology, Harbin Veterinary Research Institute of Chinese Academy of Agricultural Sciences, Harbin 150001, China; lichenxihsy@126.com (C.L.); luke0871@aliyun.com (W.S.); baixiaofei_1@163.com (X.B.); zhangqingshan91@126.com (Q.Z.); huaronghong@163.com (R.H.); liuming04@126.com (M.L.); 2 School of Electrical Engineering & Automation, Harbin Institute of Technology, Harbin 150006, China; junyanliu@126.com; 3 Institute of Genomics and Bioinformatics, National Chung Hsing University, Taichung 402, Taiwan

**Keywords:** duck Tembusu virus, E protein epitopes, type specific and cross-reactive epitopes, E protein 3D structure, diagnosis

## Abstract

Duck Tembusu virus (DTMUV) causes substantial egg drop disease. DTMUV was first identified in China and rapidly spread to Malaysia and Thailand. The antigenicity of the DTMUV E protein has not yet been characterized. Here, we investigated antigenic sites on the E protein using the non-neutralizing monoclonal antibodies (mAbs) 1F3 and 1A5. Two minimal epitopes were mapped to ^221^LD/NLPW^225^ and ^87^YAEYI^91^ by using phage display and mutagenesis. DTMUV-positive duck sera reacted with the epitopes, thus indicating the importance of the minimal amino acids of the epitopes for antibody-epitope binding. The performance of the dot blotting assay with the corresponding positive sera indicated that YAEYI was DTMUV type-specific, whereas ^221^LD/NLPW^225^ was a cross-reactive epitope for West Nile virus (WNV), dengue virus (DENV), and Japanese encephalitis virus (JEV) and corresponded to conserved and variable amino acid sequences among these strains. The structure model of the E protein revealed that YAEYI and LD/NLPW were located on domain (D) II, which confirmed that DII might contain a type-specific non-neutralizing epitope. The YAEYI epitope-based antigen demonstrated its diagnostic potential by reacting with high specificity to serum samples obtained from DTMUV-infected ducks. Based on these observations, a YAEYI-based serological test could be used for DTMUV surveillance and could differentiate DTMUV infections from JEV or WNV infections. These findings provide new insights into the organization of epitopes on flavivirus E proteins that might be valuable for the development of epitope-based serological diagnostic tests for DTMUV.

## 1. Introduction

Flaviviruses are positive-sense RNA viruses that are classified in the genus *Flavivirus*, family *Flaviviridae* [1]. Duck Tembusu virus (DTMUV) is a newly identified flavivirus that was first isolated in southeastern China in 2010 [2] and then subsequently spread to Malaysia and Thailand [3,4]. Genomic sequencing revealed that the virus was a mosquito-borne Ntaya group flavivirus [2,5,6,7]. DTMUV-infected ducks develop devastating egg production drop disease, and multiple bird species have been suggested as DTMUV hosts [5,8,9]. Postmortem examination demonstrated that infected ducks exhibited severe ovarian hemorrhage, ovaritis, and regression. The unknown transmission routes, quick spread and zoonotic nature have raised the concern of the public concerning the potential of DTMUV as a human pathogen.

In a manner similar to that of other flaviviruses, the DTMUV genome encodes three structural proteins (C, prM/M, and E) and seven nonstructural proteins (NS1, NS2A, NS2B, NS3, NS4A, NS4B, and NS5) [1,5,10]. Flavivirus structural proteins are reportedly involved in cellular attachment, membrane fusion and virion assembly, whereas the nonstructural proteins are responsible for genome replication [11]. The glycosylated E protein is located on the virion surface in most flaviviruses and plays an important role in virulence, antigenicity, host range, and tissue tropism [12,13,14]. The flavivirus E protein consists of three structurally distinct domains (D): DI, DII, and DIII. DI contains predominantly type-specific non-neutralizing epitopes [15]. DII is involved in virus-mediated membrane fusion and contains many cross-reactive epitopes that elicit neutralizing and non-neutralizing antibodies [16]. DIII contains multiple type- and subtype-specific epitopes that elicit only virus neutralizing antibodies [15,16,17]. 

Birds are the natural reservoirs or amplifying hosts for some flaviviruses, such as West Nile virus (WNV) and Japanese encephalitis virus (JEV). Laboratory diagnosis of WNV and JEV infection is predominantly serological [18,19], but caution is advised due to the high degree of cross-reactivity among flaviviruses [20,21]. An epitope-blocking enzyme immunoassay has been successfully used for the detection of virus-specific antibodies in bird serum samples [22]. Therefore, serotype-specific B cell epitopes should be identified and used to diagnose DTMUV infections in birds or to differentiate DTMUV from other flaviviruses.

In this study, we identified two E protein epitopes and assessed their cross-reactivity to other flaviviruses and their localization on the E protein 3D structure. These findings will extend our understanding of the structure-function relationships and the cross-reaction functions in the immune response. Moreover, our results provide insights into the improvement of the flavivirus serodiagnosis and the understanding of the viral pathogenesis.

## 2. Materials and Methods

### 2.1. Virus, E-Specific Monoclonal Antibodies, and JEV-, DENV-, and WNV-Positive Sera

DTMUV TA strain was grown on duck embryo fibroblasts (DEF) or embryonated eggs as previously described [6]. The E-specific monoclonal antibodies (mAbs) 1F3 and 1A5 were developed in our lab and characterized previously [23]. JEV and WNV-positive rabbit sera were donated by Dr. Ronghong Hua, (Harbin Veterinary Research Institute of Chinese Academy of Agricultural Sciences (CAAS), Harbin, China) and DENV-positive sera was donated by Dr. Xian Qi (Nanjing Municipal Centers for Disease Control and Prevention, Nanjing, China). 

### 2.2. Affinity Purification of Monoclonal Antibodies

The mAbs were purified from mouse ascites fluid using Protein G Agarose (Invitrogen, Carlsbad, CA, USA) according to the manufacturer’s instructions. The purified immunoglobulin (Ig) G antibody concentrations were determined by measuring the absorbance at 278 nm.

### 2.3. Epitope Mapping

The epitopes were mapped with purified 1A5 and 1F3 using the Ph.D-12™ Phage Display Peptide Library Kit (New England BioLabs Inc., Ipswich, MA, USA) as previously described [24,25]. Briefly, each well of a 96-well plate was coated with 10 μg/mL of purified mAb and then blocked with blocking buffer. The phage library was added to the plate and incubated for 1 h. After five washes with Tris-buffered saline (TBS) (50 mM Tris-HCl, 150 mM NaCl; pH 7.5), 1 M Tris-HCl (pH 9.1) was added to the plate [24,25]. The eluted phages were amplified and titered on lysogeny broth (LB)/isopropyl β-d-1-thiogalactopyranoside (IPTG)/5-bromo-4-chloro-3-indolyl-d-galactoside (X-Gal) plates for selection. Three rounds of biopanning were performed. The ratio of output to input was calculated as the titer of the amplified output phages/the titer of the input phages.

### 2.4. Phage Enzyme-Linked Immunosorbent Assay and Phage Clone Sequencing

After the three rounds of biopanning described above and elsewhere [24,25], individual phage clones were selected for target binding in the enzyme-linked immunosorbent assay (ELISA). Briefly, 96-well plates were coated with 100 ng of the mAbs (1F3 and 1A5) or an anti-porcine interferon (IFN)-c mAb (Sigma, St. Louis, MO, USA) as a negative control. After the coated wells were blocked, the phages (10^10^ pfu/100 μL/well) were added. The coated plates were washed ten times with phosphate buffered saline (PBS) + 0.5% (*v*/*v*) Tween-20 (PBST), and the bound phages were reacted with an horseradish peroxidase (HRP)-conjugated sheep anti-M13 antibody (Pharmacia, Piscataway, NY, USA) as previously described [24,25]. Colored precipitation was achieved by adding substrate solution containing *o*-phenylenediamine (OPD). The positive phage clones were sequenced using a previously reported sequencing primer [24,25].

### 2.5. Identification of the Essential Amino Acids in the Epitopes by Dot Blotting and Western Blot Analysis

To precisely define the epitopes, we designed and synthesized two groups of fragments corresponding to the roughly mapped epitopes. Complementary oligonucleotide primers specific for each peptide fragment were designed as previously described [26]. Nucleotide segments with Eco RI/Xho I site sticky ends were produced after direct annealing and then cloned into the pGEX6p-1 vector (GE Healthcare, Beijing, China) as previously described [26]. The expressed truncated fragments were purified using the Glutathione S-transferase (GST) Purification Kit (TaKaRa, Dalian, China). Dot blotting was performed by spotting purified peptide solution onto a nitrocellulose (NC) membrane (Millipore, Bedford, MA, USA) Approximately 1 μg of purified synthesized peptide or the unrelated control peptide YIRTPACWD (from the duck reovirus σB protein) [26] diluted with Tris sodium chloride EDTA buffer (TNE) (10 mM NaCl, 10 mM Tris-Hcl,1 mM EDTA; pH 7.4) was spotted onto the NC membrane. Then, the NC membrane was incubated with the mAbs (diluted 1:2000 in PBS) at 37 °C for 1 h. After three washes with PBST, the NC was probed with a 1:500 dilution of an HRP-conjugated goat anti-mouse IgG (KPL, Gaithersburg, MD, USA) at 37 °C for 1 h. Western blot was performed by subjecting the purified GST peptides to electrophoresis in 10% acrylamide gels, followed by electro-transfer to a NC. The membrane was probed with a duck anti-DTMUV antibody diluted 1:150 in PBST, followed by a reaction with a horseradish peroxide-conjugated goat anti-duck antibody (1:500) (KPL) for 90 min at room temperature. 

### 2.6. Sequence Analysis

To assess the level of conservation of the epitopes among the DTMUVs and other representative flaviviruses, we constructed a sequence alignment of the epitopes and determined the corresponding locations in the E proteins of the DTMUV strains and other flaviviruses using the DNASTAR Lasergene program (DNASTAR Inc., Madison, WI, USA) [27].

### 2.7. Cross-Reactivity of the Epitopes to WNV-, JEV-, and DENV-Positive Sera

Mapped epitope cross-reactions with other flaviviruses were determined by the dot blotting assay as described above. Briefly, approximately 1 μg of each synthesized epitope peptide or the control peptide YIRTPACWD diluted with TNE buffer was spotted onto the NC. Then, the NC membrane was incubated with WNV-, JEV-, and DENV-positive sera at 37 °C for 1 h. After three washes with PBST, the NC membrane was probed with a HRP-conjugated antibody targeting the corresponding IgG (KPL) at 37 °C for 1 h.

### 2.8. Protein E Modeling and Prediction

To analyze the epitope locations, we built a structure model of the DTMUV E protein. Because the DTMUV E protein shared 62% sequence identity with the JEV E protein, we chose the JEV E protein crystal structure (PDB ID: 3P54) [28] as the modeling template using MODELLE [29]. ProSA [30] and PROCHECK [31] were used to validate the stereochemical qualities of the final model. GlycoEP [32] and NGlycPred [33] were used to predict the *N*-glycosylation sites on the DTMUV E protein. The final structure was visualized and analyzed with PyMOL [34].

### 2.9. Competitive Inhibition Binding Assay of Monoclonal Antibody 1A5 to a Synthetic Peptide

To test for synthetic peptide inhibition of mAb 1A5 binding to the E protein, 100 μL of E antigen (10 μg/mL) was used to coat 96-well plates at 4 °C overnight. Then, the plates were blocked with 1% bovine serum albumin (BSA) as previously described. The peptides were synthesized in the multiple-antigen peptide (MAP) form [35]. YAEYI (in final peptide concentrations of 0, 10, 20, 40, 80, 160, and 640 μg/mL) or the unrelated control peptide YIRTPACWD was mixed with the blocking mAb 1A5 (0.2 μg/mL diluted in PBST) and incubated at room temperature for 45 min; these peptide/antibody mixtures were added to the E antigen-coated 96-well plates and incubated at room temperature for 1 h. After the plate was washed with PBST, HRP-conjugated goat anti-mouse IgG was added and the binding was assessed. The mean optical density at 405 nm (OD405) plus three times the standard deviation was used to determine the cutoff value.

### 2.10. Detection of DTMUV Infection in Duck Serum Samples

ELISA plates were coated with 50 µL/well of 640 µg/mL of synthetic peptide antigen and incubated at 4 °C overnight. After washing with PBST, the plates were blocked with PBST containing 5% (*w*/*v*) skimmed milk for 1 h at 37 °C. The diluted duck DTMUV-positive/negative sera and the WNV- and JEV-positive sera diluted in blocking solution were added and incubated for 1 h at 37 °C. After washing, 100 µL/well of the diluted goat anti-duck IgG conjugate (1:400 dilution) in blocking solution was added and incubated for 1 h at 37 °C. The plates were washed three times and then incubated with 50 µL of *p*-nitrophenyl phosphate (PNPP) substrate (Shanghai Biomedicine, Shanghai, China) for 20 min. The reactions were stopped by adding 3 M NaOH, and the plate was read on a microplate reader (Bio-Rad, Beijing, China) at 405 nm. An aliquot (100 µL/well) of the 1:400 diluted conjugate was added after washing.

## 3. Results

### 3.1. Epitope Prediction

**Figure 1:**
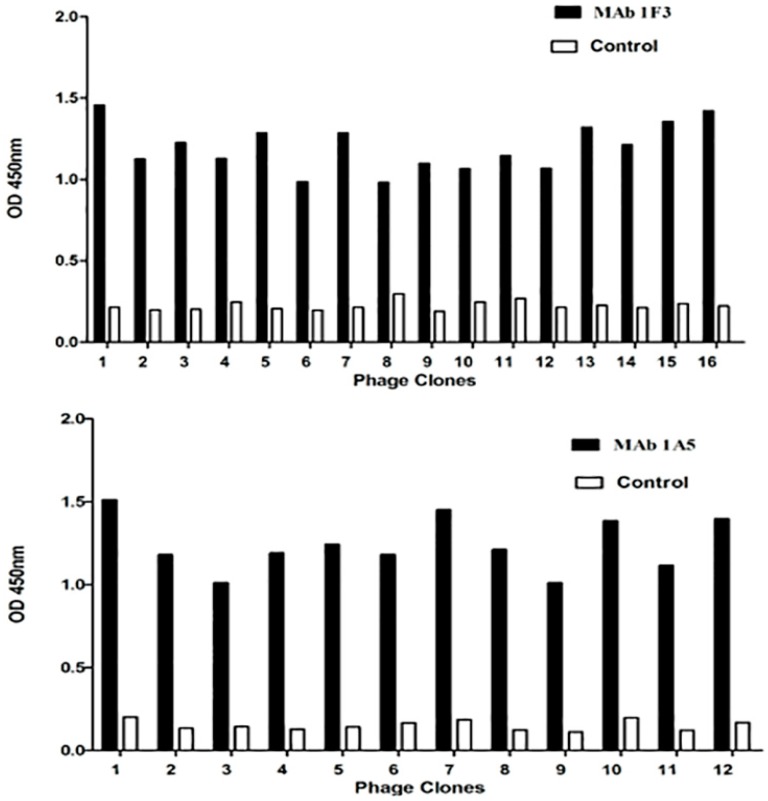
Detection of selected phages for monoclonal antibody (mAb) binding in the phage enzyme-linked immunosorbent assay (ELISA). The selected phage clones were detected by 1F3 and 1A5 or the anti-porcine interferon (IFN)-c mAb (negative control) after three rounds of biopanning. OD, optical density.

To map the locations of the E epitopes, we screened a phage-displayed 12-mer random peptide library using mAbs. After three rounds of biopanning, phage clones were selected and their reactivity with the mAbs and the negative control anti-porcine IFN-c mAb was evaluated. Sixteen of twenty-one clones (A1–A16) reacted with mAb 1F3, and 12 of 15 clones (B1–B12) reacted with 1A5 (OD450 nm ≥ 1.20); the other clones were less reactive with 1F3 and 1A5 (OD450 nm < 0.36), respectively. None of the selected clones reacted with the anti-porcine IFN-c mAb (OD450 nm < 0.27) (Figure 1). Sequencing of the phage clones with high OD values revealed the consensus sequences DLD/NLPWT (mapped with 1F3) and YAEYI (mapped with 1A5) (Table 1). These amino acid sequences are identical to the DLNLPWT (aa 220 to 226) and YAEYI (aa 87 to 91) sequences of the DTMUV TA strain E protein. 

### 3.2. Mapping of the Minimal Epitopes by Dot Blotting and Western Blot

**Figure 2:**
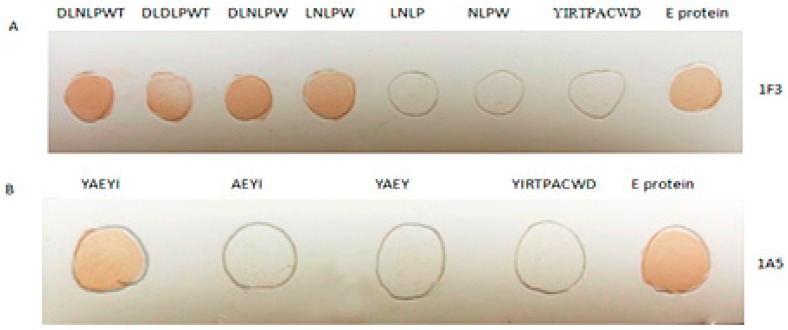
Identification of the E protein epitopes based on mAbs IF3 (**A**) and 1A5 (**B**) reactivity with the synthesized peptides in the dot blotting assay. YIRTPACWD and the E protein were used as the negative and positive control, respectively.

**Figure 3:**
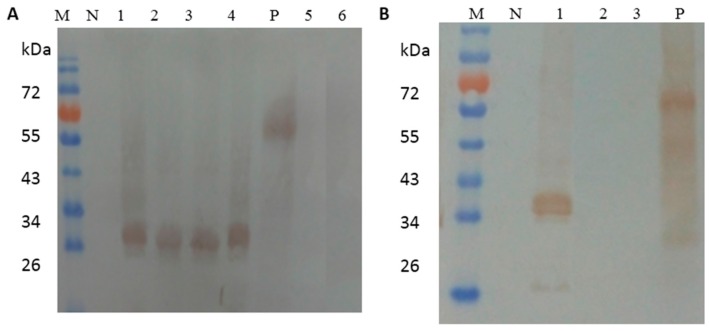
The reactivity of the synthesized mutations of the LD/NLPW (**A**) and YAEYI (**B**) peptides to duck anti-duck Tembusu virus (DTMUV) serum by Western blot. YIRTPACWD and the E protein were used as the negative (N) and positive (P) controls, respectively. M: Protein marker; N: GST negative control; (**A**) lane 1, GST-DLNLPWT; lane 2, GST-DLDLPWT; lane 3, GST-DLNLPW; lane 4, GST-LNLPW; lane 5, GST-LNLP; lane 6, NLPW; (**B**) lane 1, GST-YAEYI; lane 2, GST-AEYI; lane 3, GST-YAEY.

To confirm that the identified epitopes were recognized by the corresponding 1F3 and 1A5 mAbs, we expressed and purified fragments representing DLD/NLPWT and YAEYI (Table 2). Dot blotting showed that the DLD/NLPWT and YAEYI E protein fragments were recognized by the 1F3 and 1A5 mAbs, respectively (Figure 2A,B), whereas the control peptide YIRTPACWD did not react with the mAbs. This result suggests that the DLD/NLPWT and YAEYI fragments may be B cell epitopes of the DTMUV E protein. To define the epitopes more precisely, we synthesized substitutions or C- or N-terminal deletion mutants of the DLD/NLPWT and YAEYI peptides (Table 2). The amino acid substitution of ^222^N with ^222^D in ^220^DLD/NLPWT^226^ did not abolish the 1F3 antibody binding activity (Figure 2A), suggesting that ^222^D or ^222^N in ^220^DLD/NLPWT^226^ were mutual replicable amino acids in this position. Deletion of the amino acid ^220^D or ^226^T at the N- or C-terminus of ^220^DLNLPWT^226^ did not affect 1F3 antibody binding activity but deletion of the amino acid ^225^W abolished 1F3 binding activity, suggesting that LD/NLPW was the minimal epitope mapped by 1F3. Deletion of the amino acid ^87^Y or ^91^I at the N- or C-terminus of ^87^YAEYI^91^ abolished 1A5 binding activity, which suggested that YAEYI was the minimal epitope recognized by 1A5 (Figure 2B). The identified minimal epitopes LD/NLPW (Figure 3A) and YAEYI (Figure 3B) were confirmed by Western blot analysis with duck DTMUV-positive serum.

### 3.3. Sequence Analysis of the Identified Epitopes among the DTMUV Strains and Other Flaviviruses

**Figure 4:**
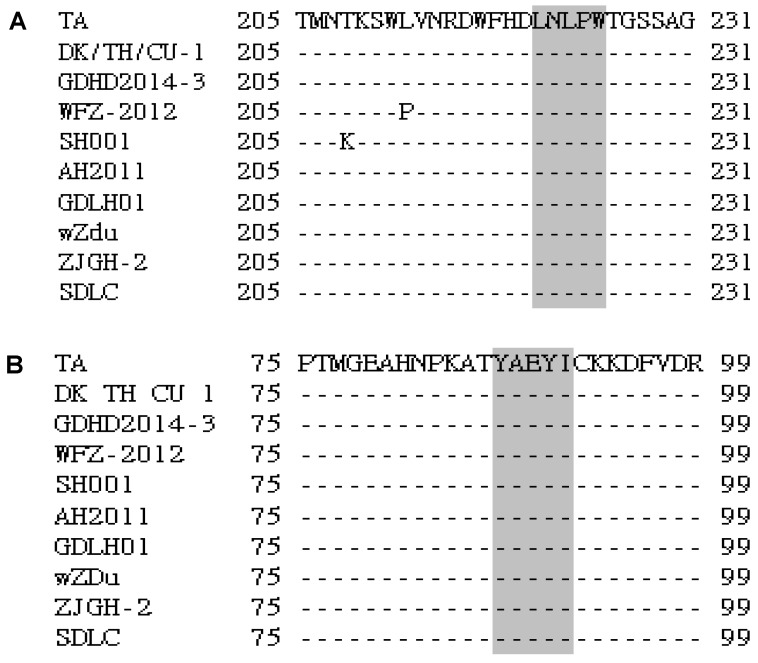
Sequence alignment of the epitope-coding regions LNLPW (**A**) and YAEYI (**B**) in the DTMUV strain E proteins. The amino acid positions for each sequence are numbered on both sides. The DTMUV TA strain sequence is shown at the top; the dashes indicate identical amino acids. The identified epitope region is boxed in grey.

**Figure 5:**
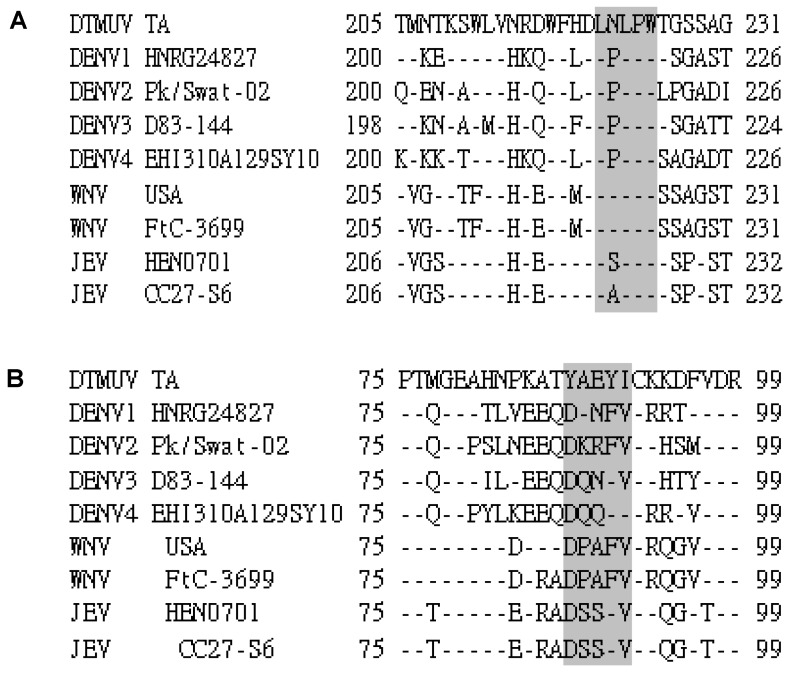
Sequence alignment of the epitope-coding regions LNLPW (**A**) and YAEYI (**B**) in the flavivirus strain E proteins. The amino acid positions for each sequence are numbered on both sides. The DTMUV TA strain sequence is shown at the top; the dashes indicate identical amino acids. The identified epitope region is boxed in grey.

To determine the conservation of LNLPW and YAEYI in the E proteins, we aligned the epitope region sequences of DTMUV with sequences from other flaviviruses. Information for the DTMUV, WNV, DENV, and JEV E protein sequences obtained from GenBank is provided in Table 3. This sequence alignment revealed that the amino acids in the ^221^LDLPW^225^ (Figure 4A) and ^87^YAEYI^91^ (Figure 4B) epitope regions were identical among the DTMUV strains, indicating that these motifs represented conserved epitopes in the DTMUV E protein. LNLPW was conserved in both DTMUV and WNV (Figure 5A) but was divergent compared to DENV and JEV. The YAEYI epitope was completely conserved in the DTMUV species but was highly divergent compared to the WNV, DENV, and JEV E protein sequences (Figure 5B), suggesting that YAEYI is a DTMUV type-specific epitope.

### 3.4. YAEYI and LNLPW Peptide Fragment Reactivity to WNV-, JEV-, and DENV-Positive Sera

**Figure 6:**
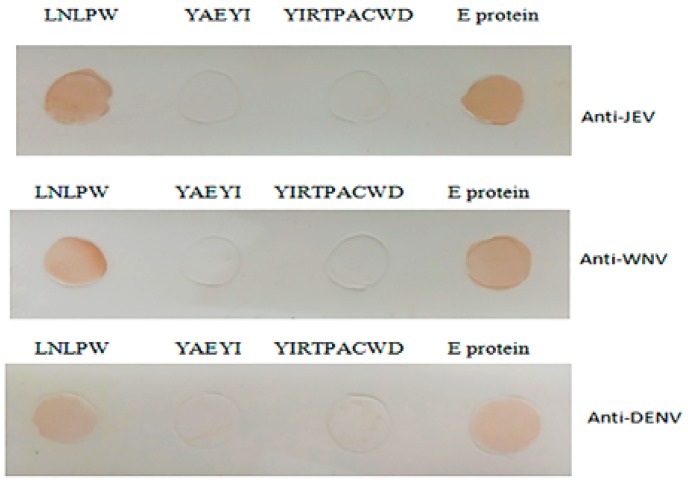
The cross-reactivity of the epitopes to the Japanese encephalitis virus (JEV)-, West Nile virus (WNV)-, and dengue virus (DENV)-positive sera in the dot blotting assay. YIRTPACWD and the E protein were used as the negative and positive controls, respectively.

To demonstrate the epitope cross-reactivity with other flaviviruses, purified YAEYI and LNLPW fragments were used to test their cross-reactivity with WNV-, JEV-, and DENV-positive sera in the dot blotting assay. The WNV-, JEV-, and DENV-positive sera reacted with the LNLPW peptide and E protein but did not react with YAEYI and the negative control peptide (Figure 6).

### 3.5. Location of Two Epitopes on the E Protein 3D Structure

**Figure 7:**
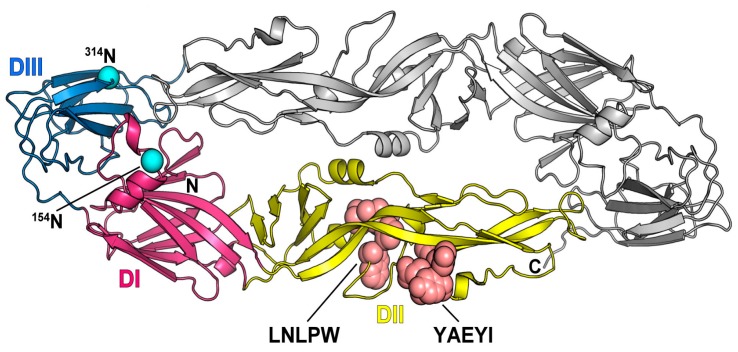
The locations of epitopes on the DTMUV E protein dimer. The DTMUV E protein structure is modeled based on the JEV E crystal structure protein using MODELLER [34]. Domains I, II and III in are colored in magenta, yellow and blue, respectively, in one monomer. The other monomer is colored grey. The locations of the two epitopes are depicted as spheres and labeled. The locations of two epitopes are depicted as spheres and labeled. Two predicted *N*-glycosylation sites by GlycoEP [32] and NGlycPred [33] are colored as cyan.

The resulting structure was evaluated by ProSA [30] and PROCHECK [31] to determine the stereochemical quality. These validation results revealed that the model structure of the DTMUV E protein shown in Figure S1 was reliable for further study. The overall structure of the DTMUV E protein resembled the structure of the previously reported flavivirus E protein [28] and had three distinct domains: a central β-barrel (domain 1), an elongated finger-like structure (domain II), and a C-terminal immunoglobulin-like module (domain III) (Figure 7). Based on the protein sequence, GlycoEP [32] suggested that the DTMUV E protein might have two potential *N*-glycosylation sites (^154^N and ^314^N), with prediction scores of 0.838 and 0.438, respectively. However, according to the structural and residue pattern information, NGlycPred [33] predicted ^154^N as the only glycosylation site with a score of 0.427. Therefore, the possibility of ^314^N serving as a glycosylation is questionable. The 3D structure of the E protein showed that two epitopes possessed loop conformations (Figure 7). Epitope YAEYI was located near the lateral ridge of domain II, and LNLPW was located close to the domain II central interface.

### 3.6. Competitive Inhibition of Synthetic Peptide YAEYI Binding to Monoclonal Antibody 1A5

**Figure 8:**
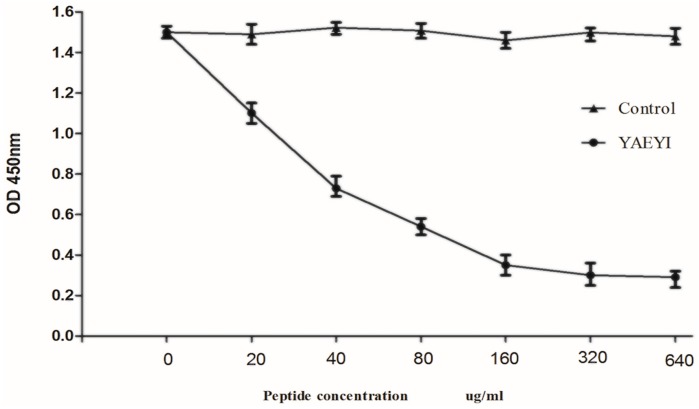
Competitive inhibition of synthetic peptide YAEYI binding to mAb 1F3. A competitive ELISA was performed using the antigen peptide YAEYI as the competitor for the E protein. Values represent three independent experiments with triplicate determinations included in each experiment (*p* < 0.05, Student’s *t*-test).

The peptides were synthesized in the MAP form because the binding efficiency of an eight-chain MAP is greater than the binding efficiency of a single-chain peptide [35]. Competitive binding assays were performed to confirm that the E protein peptide YAEYI was an E protein epitope. These assays showed that the reactivity of mAb 1A5 with the E protein was markedly inhibited by the synthetic antigen peptide YAEYI in a dose-dependent manner (*p* < 0.05; Figure 8) after Student’s *t*-test statistical method analysis.

### 3.7. Sensitivity and Reactivity of an Epitope-Based Peptide Applied for the Diagnosis of DTMUV in Serum Samples

**Figure 9:**
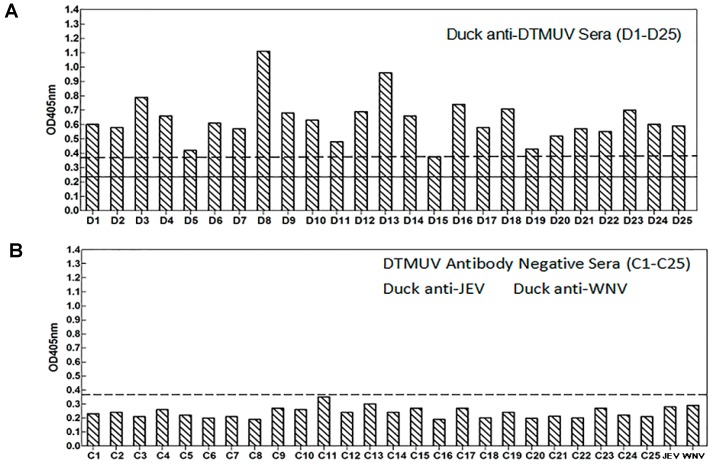
ELISA reactivity of the DTMUV synthetic peptide against serum samples from 25 DTMUV-infected duck (**A**) versus sera from 25 healthy ducks (**B**). The cutoff value (dashed lines) was calculated as 0.375. Solid line, mean OD405.

Mean optical density at 405 nm (0.235) plus three times the standard deviation (0.0465) was used to determine the cutoff value (0.375). The synthetic antigen peptide YAEYI was able to detect DTMUV infections in 24 serum samples collected from 25 DTMUV infections confirmed by ELISA (Figure 9). One serum (D15) was detected negative by peptide YAEYI (0.374). In contrast, 25 SPF duck sera, anti-WNV sera, and anti-JEV sera were seronegative using the same epitope-based peptide serologic test. The specificity of this test was 100% for the SPF duck sera without DTMUV infection (Figure 9). The sensitivity of this epitope-based peptide serologic test for DTMUV infection was 96%.

## 4. Discussion

Monoclonal antibodies against flaviviruses are powerful tools for mapping flavivirus epitopes and investigating antigenic structures. The flavivirus E protein was confirmed to be a strong immunogen for antibody production. The aims of this study were to investigate the antigenic sites on the DTMUV structural envelop E protein using mAbs against E protein fragments and to define the B cell epitopes recognized by these antibodies. Epitopes for two mAbs were mapped precisely to clusters in two locations (i.e., ^220^DLD/NLPWT^226^ (1F3) and ^87^YAEYI^91^ (1A5)) on the E protein using a 12-mer random peptide phage display system. The dot blotting assay with mAbs and deleted peptide fragments from each group showed the minimal epitopes for IF3 and 1A5 on LNLPW and YAEYI. Anti-DTMUV duck sera recognized the two epitopes in the Western blot assay, indicating the importance of the minimal antigenic domains of the epitopes for antibody-epitope binding reactivity.

The sequence alignment revealed that epitope YAEYI was completely conserved among the various DTMUV strains but was highly divergent from the WNV, DENV, and JEV strains, which suggested the YAEYI was a DTMUV type-specific epitope mapped by mAb 1A5. This domain did not exhibit cross-reactivity with WNV, DENV, and JEV, suggesting that it might have an application for the specific serological detection of DTMUV infection. Moreover, the structure model of the DTMUV E protein showed that the YAEYI epitope was near the lateral ridge of DII and protruded from the surface of the E protein, which supported the hypothesis that the YAEYI peptide could be easily exposed and used as an antigen to detect DTMUV type-specific infection. The mAb 1A5 is expected to react broadly with various DTMUV strains; however, the YAEYI-based ELISA reactivity for the detection of DTMUV-infected duck serum samples was not as high as the detection obtained using whole virus or viral proteins, which had multiple epitopes. Therefore, identifying more DTMUV-specific epitopes and combining more epitope-based peptide antigens for DTMUV-positive sera detection will increase the sensitivity of this serologic diagnosis method. Using this epitope-based peptide antigen to detect DTMUV-positive sera is relatively simple and specific and does not require the paired serum samples needed for conventional tests. Furthermore, the WNV-/JEV-positive serum samples showed no ELISA reactivity with the YAEYI-based antigen, which suggested that the assay was capable of differentiating between DTMUV and WNV/JEV in duck serum samples. Further study on the application of this YAEYI-based ELISA with a large number of samples is in progress. 

A previous report showed that the JEV epitope reacted with both JEV-positive and WNV-positive sera [36], suggesting that JEV and WNV were two members of the JEV serocomplex; this association caused the cross-reactivity among these flaviviruses. The sequence alignment showed that the LNLPW epitope was highly conserved in both DTMUV and WNV, indicating that DTMUV and WNV contained the same immunodominant epitope. This hypothesis was confirmed by the dot blotting assay, which showed that anti-WVN sera could bind LNLPW, suggesting that DTMUV and WNV were also part of a serocomplex. 

The 3D structure of the E protein suggested that the LNLPW epitope did not protrude from the E protein surface. Previous studies in the DENV virion particle demonstrated that the binding of some E reactive antibodies relied on the dynamic motion of protein molecules (“breathing”), leading to transient exposure of buried epitopes [37,38,39]. Accordingly, whether the “breathing” of the DTMUV E protein will expose the two epitopes and allow mAb binding or inhibit the activity remains to be experimentally resolved.

## 5. Conclusions

In summary, we identified for the first time two novel epitopes of the DTMUV E protein: one DTMUV type-specific epitope and one widely cross-reactive epitope. We also reported for the first time the 3D structure of the E protein and epitope locations. This information has provided new insights into the structure and organization of epitopes on the DTMUV E protein and valuable epitope information for the development of diagnostic assays for the specific detection of DTMUV infection.

## Figures and Tables

**Table 1:** Peptide sequences of the selected phage clones.

Phage Clone	Sequence																		Phage Clone	Sequence														
A1						E	L	N	**L**	**P**	**W**	Q	R	N	A	L	V		B1	S	R	N	L	S	**Y**	**A**	**E**	**Y**	**I**	Q	I			
A2		S	A	E	N	**D**	**L**	T	**L**	**P**	**W**	**T**	T						B2				G	N	**Y**	S	**E**	**Y**	**I**	V	G	K	L	V
A3		M	A	N	A	E	I	**D**	**L**	**P**	**W**	**T**	K						B3				S	S	**Y**	**A**	N	**Y**	**I**	Q	F	R	N	T
A4			H	P	H	**D**	**L**	**N**	D	L	T	S	P	F					B4				S	S	**Y**	T	A	**Y**	**I**	M	A	R	G	Q
A5		E	F	W	T	A	**L**	S	D	**P**	**W**	Y	F						B5	N	S	M	S	E	**Y**	I	N	**Y**	**I**	L	T			
A6					A	H	**L**	H	D	**P**	F	**T**	T	L	S	P			B6				V	D	**Y**	S	T	**Y**	**I**	S	R	L	T	S
A7		L	D	F	H	**D**	**L**	N	R	**P**	F	N	N						B7		N	F	M	N	**Y**	**A**	**E**	**Y**	V	Q	K	K		
A8			T	H	D	P	**L**	**D**	S	**P**	**W**	N	F	S					B8				V	D	**Y**	S	T	**Y**	**I**	S	R	L	T	S
A9				F	N	**D**	**L**	**D**	**L**	**P**	F	G	K	R	A				B9		T	V	H	S	**Y**	E	**E**	**Y**	T	A	R	R		
A10				S	Y	**D**	**L**	**D**	**L**	**P**	**W**	I	A	R	K				B10			V	S	P	**Y**	**A**	**E**	**Y**	W	L	S	Q	M	
A11			S	F	L	E	**L**	**D**	**P**	**P**	**W**	**T**	T	N					B11				W	D	**Y**	N	L	**Y**	**I**	K	Y	V	A	R
A12			Q	H	S	F	**L**	**D**	**L**	**P**	**W**	H	L	T					B12				V	D	**Y**	**A**	T	**Y**	**I**	S	R	L	T	S
A13			H	P	H	**D**	**L**	**N**	**L**	**P**	T	S	P	F																				
A14			H	P	H	**D**	**L**	**N**	**L**	**P**	T	S	P	F																				
A15		M	A	N	A	**D**	**L**	**N**	**L**	**P**	**W**	**T**	K																					
A16	T	S	H	S	W	**D**	**L**	**N**	**L**	**P**	S	G																						
Consensus						**D**	**L**	**D/N**	**L**	**P**	**W**	**T**													**Y**	**A**	**E**	**Y**	**I**					
Virus TA			219		H	**D**	**L**	**N**	**L**	**P**	**W**	**T**	226							84		K	A	T	**Y**	**A**	**E**	**Y**	**I**	C	K	K	D	97

Consensus amino acids are shown in bold.

**Table 2:** Primers for the truncated epitope fragments.

Primers	Sequence	Truncated Peptide
1F3-1-F	5′-aattcgatctcaacttaccatggacac-3′	GST-DLNLPWT
1F3-1-R	5′-tcgagtgtccatggtaagttgagatcg-3′	
1F3-2-F	5′-aattcgatctcgacttaccatggacac-3′	GST-DLDLPWT
1F3-2-R	5′-tcgagtgtccatggtaagtcgagatcg-3′	
1F3-3-F	5′-aattcgatctcaacttaccatggc-3′	GST-DLNLPW
1F3-3-R	5′-tcgagccatggtaagttgagatcg-3′	
1F3-4-F	5′-aattcctcaacttaccatggc-3′	GST-LNLPW
1F3-4-R	5′-tcgagccatggtaagttgagg-3′	
1F3-5-F	5′-aattcctcaacttaccac-3′	GST-LNLP
1F3-5-R	5′-tcgagtggtaagttgagg-3′	
1F3-6-F	5′-aattcaacttaccatggc-3′	GST-NLPW
1F3-6-R	5′-tcgagccatggtaagttg-3′	
1A5-1-F	5′-aattctacgctgaatacatac-3′	GST-YAEYI
1A5-1-R	5′-tcgagtatgtattcagcgtag-3′	
1A5-2-F	5′-aattcgctgaatacatac-3′	GST-AEYI
1A5-2-R	5′-tcgagtatgtattcagcg-3′	
1A5-3-F	5′-aattctacgctgaatacc-3′	GST-YAEY
1A5-3-R	5′-tcgaggtattcagcgtag-3′	

GST, Glutathione S-transferase.

**Table 3:** Flavivirus strains used in the sequence analysis in this study.

Species	Strain	GenBank No.	Location/Year of Isolation	
DTMUV	TA	JQ289550.1	China	2010
DTMUV	DK/TH/CU-1	KR061333.1	Thailand	2013
DTMUV	GDHD2014-3	KT159713.1	China	2014
DTMUV	WFZ-2012	KC990545.1	China	2012
DTMUV	SH001	KP742476.1	China	2015
DTMUV	AH2011	KJ958533.1	China	2012
DTMUV	GDLH01	KT824876.1	China	2015
DTMUV	WZDu	AB917089.1	China	2012
DTMUV	ZJ GH-2	JQ314465.1	China	2010
DTMUV	SDLC	KJ740747.1	China	2013
DENV-1	HNRG24827	KC692511.1	Argentina	2010
DENV-2	DENV-2/Pk/Swat-02	KJ701507.1	Pakistan	2013
DENV-3	D83-144	KJ737430.1	Thailand	1983
DENV-4	EHI310A129SY10	JX024758.1	Singapore	2010
WNV	USA	AY646354.1	USA	2002
WNV	FtC-3699	KR868734.1	USA	2012
JEV	HEN0701	FJ495189.1	China	2007
JEV	CC27-S6	AY303797.1	Taiwan	2003

DTMUV, duck Tembusu virus; DENV, dengue virus; WNV, West Nile virus; JEV, Japanese encephalitis virus.

## Supplementary Materials

The following are available online at www.mdpi.com/1999-4915/8/11/306/s1, Figure S1: The stereochemical quality of the structure evaluated by ProSA and PROCHECK.
