# Supplementary material for: Epitope Identification and Application for Diagnosis of Duck Tembusu Virus Infections in Ducks
Source: Viruses. 2016 Nov 10;8(11):306. doi: 10.3390/v8110306 (PMC5127020; doi:10.3390/v8110306)
Supplement: Supplementary file 1 [file viruses-08-00306-s001.docx]

Supplementary Materials: Epitope Identification and Application for Diagnosis of Duck Tembusu Virus Infections in Ducks

Chenxi Li, Junyan Liu, Wulin Shaozhou, Xiaofei Bai, Qingshan Zhang, Ronghong Hua,
Jyung-Hurng Liu, Ming Liu and Yun Zhang


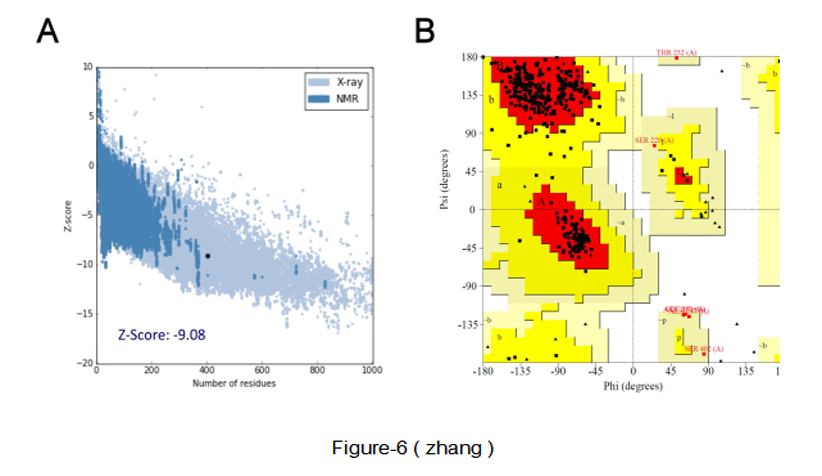


**Figure S1.** The stereochemical quality of the structure evaluated by ProSA and PROCHECK.
